# Supplementary material for: Genomic Insights into High-Altitude Adaptation: A Comparative Analysis of Roscoea alpina and R. purpurea in the Himalayas
Source: Int J Mol Sci. 2024 Feb 14;25(4):2265. doi: 10.3390/ijms25042265 (PMC10889555; doi:10.3390/ijms25042265)
Supplement: Supplementary file 1 [file ijms-25-02265-s001.zip › ijms-2813964-supplementary.pdf]

Supplemental Information

Table S1. Tajima's *D* mean value of ten random samples

Sample size	4		5		6		7	
	R. alpina	*R. purpurea*	*R. alpina*	*R. purpurea*	*R. alpina*	*R. purpurea*	*R. alpina*	*R. purpurea*
1	0.5065	0.1126	0.4177	-0.2222	0.3681	-0.2004	0.3355	-0.2004
2	0.4121	-0.0208	0.3823	-0.0817	0.3459	-0.2135	0.3355	-0.2135
3	0.4234	-0.0241	0.4780	-0.1315	0.3459	-0.2065	0.3355	-0.2065
4	0.4674	-0.0306	0.3667	-0.0278	0.3456	-0.1969	0.3355	-0.1969
5	0.5662	0.0471	0.3823	-0.1452	0.3418	-0.1215	0.3355	-0.1215
6	0.4283	0.1165	0.3827	-0.1440	0.4780	-0.2650	0.3355	-0.2650
7	0.4730	0.0782	0.5435	-0.1484	0.3459	-0.1947	0.3355	-0.1947
8	0.4932	-0.0268	0.3861	-0.0889	0.3681	-0.2113	0.3355	-0.2113
9	0.4872	0.1255	0.3688	-0.0690	0.3418	-0.1152	0.3355	-0.1152
10	0.4097	0.0938	0.3840	-0.1364	0.3479	-0.1972	0.3355	-0.1972
mean ± *SD*	0.4667 ±0.047	0.0471 ±0.063	0.4092 ±0.054	-0.1195 ±0.051	0.3629 ±0.04	-0.1922 ±0.042	0.3355 ±0.0	-0.1922 ±0.042

Table S2. Nucleotide diversity (π) mean value of ten times random sampling

Sample size	4		5		6		7	
	R. alpina	*R. purpurea*	*R. alpina*	*R. purpurea*	*R. alpina*	*R. purpurea*	*R. alpina*	*R. purpurea*
1	0.0018	0.0027	0.0018	0.0028	0.0017	0.0026	0.0017	0.0026
2	0.0019	0.0029	0.0017	0.0025	0.0017	0.0025	0.0017	0.0025
3	0.0019	0.0028	0.0017	0.0026	0.0017	0.0026	0.0017	0.0026
4	0.0020	0.0028	0.0018	0.0025	0.0017	0.0025	0.0017	0.0025
5	0.0019	0.0028	0.0017	0.0027	0.0017	0.0024	0.0017	0.0024
6	0.0019	0.0026	0.0018	0.0027	0.0016	0.0025	0.0017	0.0025
7	0.0020	0.0028	0.0017	0.0027	0.0017	0.0025	0.0017	0.0025
8	0.0018	0.0029	0.0018	0.0025	0.0017	0.0025	0.0017	0.0025
9	0.0019	0.0026	0.0018	0.0026	0.0017	0.0024	0.0017	0.0024
10	0.0018	0.0027	0.0017	0.0027	0.0017	0.0025	0.0017	0.0025
mean ± *SD*	0.0019 ±0.0	0.0028 ±0.0	0.0018 ±0.0	0.0026 ±0.0	0.0017 ±0.0	0.0025 ±0.0	0.0017 ±0.0	0.0025 ±0.0

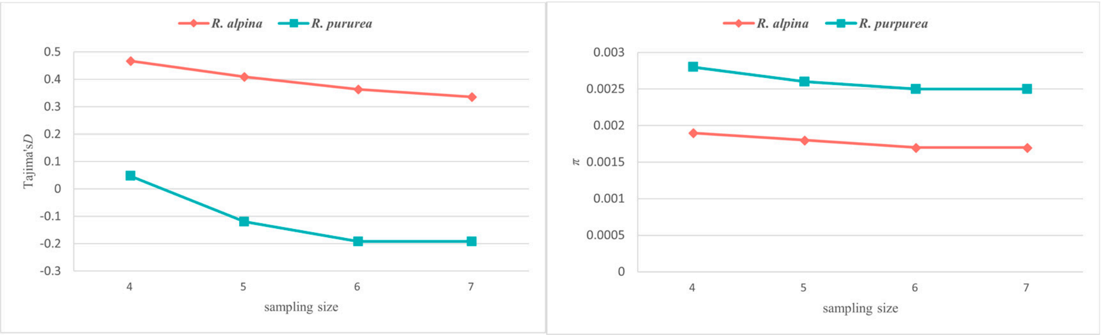

(**a**) (**b**)

Figure S1. The sampling mean value line chart of Tajima's *D* and π of different sample sizes. (a) average Tajima's D of the two species. (b) average π of the two species.

Table S3. Number of windows with F_{ST} = 1 under 10 times random sampling.

Sample size	12	11	10	9	8	7
1	150	154	160	162	150	174
2	142	148	154	171	154	163
3	149	150	156	168	158	164
4	153	154	162	161	163	165
5	153	155	147	170	155	150
6	153	145	159	169	163	173
7	150	150	163	159	168	160
8	150	159	158	166	163	159
9	144	154	156	153	163	178
10	145	159	156	163	159	160
In Common	76	76	76	80	76	76

Table S4. Number of genes with F_{ST} = 1 under 10 times random sampling.

Sample size	12	11	10	9	8	7
1	112	115	126	122	107	134
2	107	108	125	134	122	130
3	113	120	114	130	117	129
4	120	119	129	119	117	130
5	120	124	107	126	116	115
6	120	124	119	140	117	126
7	113	108	123	116	126	127
8	121	122	112	134	128	111
9	109	115	122	111	121	137
10	109	124	117	124	120	124
In common	58	58	58	61	58	58

Table S5. Gene list of genes in common under random sampling.

Gene ID	Gene symbol
evm.model.000039FARROWPILON.191	
evm.model.000361FARROWPILON.95	
evm.model.000361FARROWPILON.98	*RLK*
evm.model.000260FARROWPILON.16	
evm.model.000260FARROWPILON.15	
evm.model.000012FARROWPILON.162	
evm.model.000012FARROWPILON.165	
evm.model.000305FARROWPILON.23	
evm.model.000305FARROWPILON.19	
evm.model.000061FARROWPILON.132	
evm.model.000061FARROWPILON.134	
evm.model.000061FARROWPILON.144	
evm.model.000061FARROWPILON.133	

evm.model.000063FARROWPILON.40	*FAR1*
evm.model.000027FARROWPILON.330	
evm.model.000536FARROWPILON.1	*BRs*
evm.model.000166FARROWPILON.72	
evm.model.000005FARROWPILON.285	
evm.model.000224FARROWPILON.45	
evm.model.000258FARROWPILON.11	*CALS9*
evm.model.000258FARROWPILON.12	*SHAT1-5*
evm.model.000239FARROWPILON.49	
evm.model.000239FARROWPILON.51	*REL2*
evm.model.000018FARROWPILON.158	*E2*
evm.model.000018FARROWPILON.157	
evm.model.000367FARROWPILON.20	
evm.model.000262FARROWPILON.12	*POD*
evm.model.000179FARROWPILON.39	*AtLPP1*
evm.model.000025FARROWPILON.1	
evm.model.000042FARROWPILON.7	
evm.model.000178FARROWPILON.68	*RFS2*
evm.model.000419FARROWPILON.2	
evm.model.000419FARROWPILON.3	
evm.model.000011FARROWPILON.116	
evm.model.000413FARROWPILON.9	
evm.model.000074FARROWPILON.41	
evm.model.000035FARROWPILON.220	*PER65*
evm.model.000035FARROWPILON.218	
evm.model.000546FARROWPILON.4	
evm.model.000546FARROWPILON.10	
evm.model.000546FARROWPILON.5	
evm.model.000441FARROWPILON.4	
evm.model.000441FARROWPILON.6	
evm.model.000441FARROWPILON.5	
evm.model.000472FARROWPILON.1	*AAEs*
evm.model.000125FARROWPILON.34	*CK2*
evm.model.000014FARROWPILON.110	
evm.model.000096FARROWPILON.60	*StEXPA3*
evm.model.000156FARROWPILON.26	*RPN8a*
evm.model.000377FARROWPILON.7	*MEE40*
evm.model.000140FARROWPILON.156	
evm.model.000140FARROWPILON.157	
evm.model.000266FARROWPILON.80	
evm.model.000040FARROWPILON.173	*VAR3*
evm.model.000095FARROWPILON.67	
evm.model.000457FARROWPILON.3	
evm.model.000457FARROWPILON.1	
evm.model.000457FARROWPILON.2	

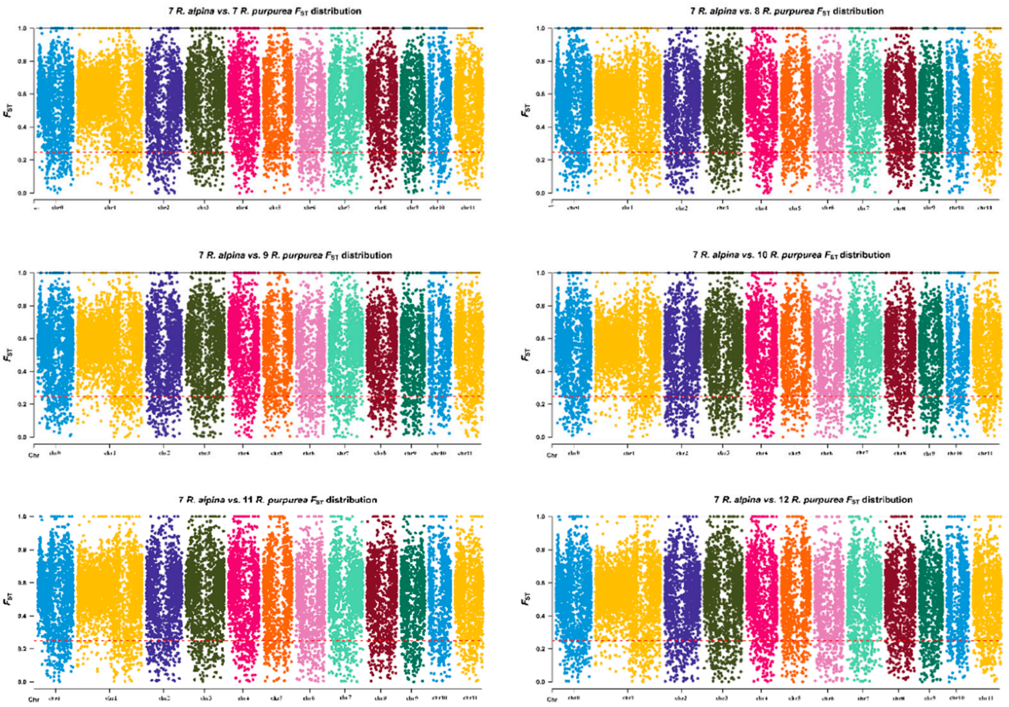

Figure S2. Manhattan plot of genome-wide F_{ST} between *R. alpina* and random sampling *R. purpurea*. The red dashed line indicates F_{ST} = 0.25 and the black solid line indicates F_{ST} = 1.

Table S6. Samples information, resequencing data size, Inbreeding coefficient (F_{IS}) and genome heterozygosity (H_E) of each individual.

Species	Sample ID	Location	Data size (Gb)	F_{IS}	H_E (%)
R. alpina	LJ2	Jilong, Tibet, China	58	0.793	0.053
	al-2	Yadong, Tibet, China	47	0.809	0.075
	Ral2	Yadong, Tibet, China	53	0.804	0.080
	Ral3	Kyanjin Gumba, Nepal	51	0.801	0.076
	R161	Jumla, Nepal	52	0.803	0.053
	R161-3	Jumla, Nepal	58	0.716	0.045
	R161-4	Jumla, Nepal	48	0.805	0.073
R. purpurea	Rnew1	Ganesh Himal, Nepal	53	0.233	1.021
	Rnew2	Ganesh Himal, Nepal	47	0.217	0.990
	Rnew3	Ganesh Himal, Nepal	46	0.206	1.129
	RpurB1	Jumla, Nepal	53	0.244	0.714
	RpurB2	Jumla, Nepal	77	0.234	0.824
	RpurR1	Ganesh Himal, Nepal	46	0.331	0.857
	RpurR2	Ganesh Himal, Nepal	47	0.323	0.824
	E1	LangTang, Nepal	56	0.261	0.766
	E2	LangTang, Nepal	59	0.255	0.826
	xpur2	Yadong, Tibet, China	53	0.261	0.938
	xpur-2	Yadong, Tibet, China	64	0.217	0.906
	xpur2-1	Nagarkot, Nepal	57	0.252	0.916
	xpurzi	Mudhe, Nepal	47	0.258	1.163
